# Antiplatelet, Antithrombotic, and Fibrinolytic Activities of *Campomanesia xanthocarpa*

**DOI:** 10.1155/2012/954748

**Published:** 2011-09-07

**Authors:** Jonatas Zeni Klafke, Mariane Arnoldi da Silva, Mateus Fortes Rossato, Gabriela Trevisan, Cristiani Isabel Banderó Walker, Cláudio Alberto Martins Leal, Diego Olschowsky Borges, Maria Rosa Chitolina Schetinger, Rafael Noal Moresco, Marta Maria Medeiros Frescura Duarte, Adair Roberto Soares dos Santos, Paulo Ricardo Nazário Viecili, Juliano Ferreira

**Affiliations:** ^1^Programa de Pós-Graduação em Ciências Biológicas: Bioquímica Toxicológica, Universidade Federal de Santa Maria, 97105-900 Santa Maria, RS, Brazil; ^2^Grupo Multidisciplinar de Saúde, Universidade de Cruz Alta, 98020-290 Cruz Alta, RS, Brazil; ^3^Programa de Pós-Graduação em Ciências Farmacêuticas, Centro de Ciências da Saúde, Universidade Federal de Santa Maria, 97105-900 Santa Maria, RS, Brazil; ^4^Departamento de Ciências da Saúde, Universidade Luterana do Brasil, 97020-001 Santa Maria, RS, Brazil; ^5^Department of Physiological Sciences, Universidade Federal de Santa Catarina, 88040-970 Florianópolis, SC, Brazil

## Abstract

In a previous work based on popular belief, *Campomanesia xanthocarpa* Berg., popularly known as “guavirova”, showed to have a potential effect in the control of a number of conditions associated with cardiovascular diseases. The aim of the present work was to investigate the effects of *C*. *xanthocarpa* extract (CXE) on antiplatelet, antithrombotic and fibrinolytic activities in mice and in human blood. Mice were treated orally for 5 days with CXE or acetylsalicylic acid and at the end of the treatment period animals were challenged for bleeding, acute thromboembolism and ulcerogenic activity. In addition, we have assessed the prothrombin time and activated partial thromboplastin time (aPTT) after oral administration. In *in vitro* assays, antiplatelet effects of CXE was evaluated on platelet aggregation, and fibrinolytic activity of the extract was observed by mice or human artificial blood clot degradation. Platelet citotoxicity of the extract was also determined by the LDH assay. Results demonstrated that CXE has a significant protective effect on thrombosis. It also inhibits platelet aggregation without demonstrating cytotoxicity on platelets. CXE slightly prolonged aPTT and showed no ulcerogenic activity after oral administration. In addition, CXE showed a fibrinolytic activity. Thus, *C*. *xanthocarpa* showed antiplatelet, antithrombotic and fibrinolytic activities in mice.

## 1. Introduction

The interaction between platelets and blood vessels is important in the development of thrombosis and cardiovascular diseases [1]. Platelets are essential in the maintenance of cardiovascular integrity and in the control of bleeding through forming blood clot. However, they are also implicated in the pathological progression of atherosclerotic lesions and arterial vascular thrombosis [2]. Uncontrolled platelet aggregation is critical in arterial thrombosis and may cause life threatening disorders [3]. Antiplatelet agents are therefore considered as a key tool in the treatment and/or prevention of cardiovascular thrombotic diseases [4]. Although it is well established that aspirin still provides an effective secondary prevention of ischemic cardiovascular disorders, this drug can produce hemorrhagic events and upper gastrointestinal bleeding as major drawbacks [5]. 

During the past decade, several trials have led to an effort in the search for novel compounds or sources to suppress the platelet aggregation [6, 7]. As a consequence, a number of protective effects of plants against the serious health risks have been summarized due to thrombotic diseases, such as coronary thrombosis and atherosclerosis, and numerous experimental studies have been carried out both *in vivo *and *in vitro *[8, 9]*.*

In Brazil, the edible plant *Campomanesia xanthocarpa *Berg. (Myrtaceae), popularly known as “guavirova,” is present in the Southern region, and is likewise found in Argentina, Paraguay, and Uruguay [10]. Studies have shown that *C. xanthocarpa *possesses a wide spectrum of physiological effects: the leaves of this plant are used as infusion in folk medicine to treat inflammatory diseases and hypercholesterolemia [11]. Moreover, *C. xanthocarpa* is empirically used for weight loss and for the control of a number of conditions associated with obesity [12]. One of the most recent studies demonstrated that the *C. xanthocarpa *produced an effect parallel with the mechanism of oral hypolipemiants, and that this plant showed intense presence of saponins [13], which are widely distributed in plants and have many biological activities, such as antiplatelet activity [14, 15]. Since hypolipemiants also exert antithrombotic effects [16, 17], and although *C. xanthocarpa* has reduced the blood cholesterol levels in hypercholesterolemic patients [13], until now no information has been available about the antithrombotic effect of *C. xanthocarpa*.

*C. xanthocarpa* has been used in the prevention and treatment of cardiovascular diseases based on popular belief and has recently received considerable attention, but the antithrombotic and fibrinolytic activities of this plant still remain unknown. Thus, the aim of the present work was to investigate the effects of *C. xanthocarpa* on antithrombotic and fibrinolytic activities in mice.

## 2. Materials and Methods

### 2.1. Drugs and Reagents

Drugs used in the present study were acetylsalicylic acid (ASA), adenosine diphosphate (ADP), epinephrine and calf collagen type III used for experiments provided from Sigma-Aldrich (St. Louis, USA). Furthermore, streptokinase used in *in vitro *experiments was obtained from Bergamo (São Paulo, Brazil).

### 2.2. Plant and Extract Preparation

Leaves were collected in May, 2010 from a *Campomanesia xanthocarpa* tree in the city of Cruz Alta, RS, Brazil. A Voucher specimen number 1088 was deposited at the Herbarium of University of Cruz Alta. The material collected underwent a cleaning process involving 1 h in a diluted solution of 20% hypochlorite made from a 2% stock solution (final concentration of hypochlorite in diluted solution of 0.4%), immediately followed by washing in running potable water for 15 min. Then, the material was dried at 40–45°C and triturated to a fine powder [13]. 

In order to carry out the tests, an extract of *C. xanthocarpa* leaves was prepared. Initially, 500 mg of dry leaves was added to 30 mL of a water solution at 37°C under constant agitation for 30 min. After, the solution was filtered and evaporated to determine total dry content. The final powder was diluted in water and then adjusted to the desired concentration to perform the tests.

### 2.3. Animal and Human Participants

Male Swiss mice (30–40 g) were used and all animals were housed under standard conditions, with constant temperature (22–24°C) and humidity (55–65%) levels, a 12 h dark-light cycle, and free access to food and water. Animals were acclimatized to the laboratory for at least 1 h before testing. The present study was conducted in accordance with current guidelines for the care of laboratory animals and all procedures were approved by our Institutional Ethics Committee of the Federal University of Santa Maria (number 80/2010). Besides, human blood was collected of six healthful individuals, with showed 30  ± 7 years of age to evaluate *in vitro* assays, and our research followed guidelines of the Declaration of Helsinki and Tokyo for humans and informed consent was obtained for all the six subjects.

### 2.4. *In Vitro* Assays

#### 2.4.1. Blood Manipulation

Blood samples were collected from mice or subjects into Vacutainer (BD Diagnostics, Plymouth, UK) tubes with sodium citrate 3.8% (1 part of citrate : 9 parts of blood). The samples of blood from mice were collected from the hepatic vein. The blood samples were centrifuged for 15 min at 180 ×g to obtain platelet-rich plasma (PRP) or 10 min at 2000 ×g to obtain platelet-poor plasma (PPP).

#### 2.4.2. Platelet Aggregation Test

To evaluate the platelet aggregation, the test was performed by turbidimetric measurement with a Chrono-log optical aggregometer, with AGGRO/LINK Model 810-CA software for Windows version 5.1 [18]. After calibration of the aggregometer, the sample data concerning the assays and reagents were entered on a computer coupled to the equipment, and the sample test was then performed. Aggregation was recorded as the percent change in light transmission: the baseline value was set using PRP and maximal transmission using PPP. PRP was preincubated at 37°C for 4 min with vehicle or the *C. xanthocarpa* before addition of the platelet agonist, adenosine diphosphate (ADP). Maximal aggregation was obtained stimulating platelet with ADP 10 *μ*M.

#### 2.4.3. Determination of Cytotoxicity

To verify the possible effect of cytotoxicity of *C. xanthocarpa* on platelets, we analyzed the leakage of lactate dehydrogenase (LDH) from platelets of human. After incubation with test drugs at 37°C for 3 min, aliquots were collected and centrifuged at room temperature for 2 min at 12.000 ×g. A 50 *μ*L aliquot of the resultant supernatant was used to measure the LDH leakage using enzymatic methods with (Labtest, Lagoa Santa-MG, Brazil). The extent of LDH leakage was expressed as % of total enzyme activity measured in platelets completely lysed with 10% sodium dodecyl sulphate (SDS final concentration was 0.05%).

#### 2.4.4. Fibrinolytic Activity

The fibrinolytic activity of the *C. xanthocarpa* extract was observed by artificial blood clot degradation [19]. An artificial blood clot was made by spontaneous coagulation of 50 *μ*L of fresh mice or human blood in a glass test tube. One hour later, the artificial blood clot was rinsed out repeatedly. The artificial blood clot was dipped in 1, 3, 10, 30, and 100 *μ*g/mL of *C. xanthocarpa *extract or streptokinase at room temperature. Saline was used as a control. The degradation of the clot was estimated by color development after 1 h at room temperature. Then, red blood cells were lysed by adding 20 *μ*L of triton 5% and then the absorbance of the final solution was read at 560 nm. The amount of color was estimated by linear regression analysis of a standard curve obtained from samples of mice blood properly diluted.

### 2.5. *In Vivo* Assays

#### 2.5.1. Animal Treatment

The mice were randomly assigned to distinct groups subjected to the following treatments: 10, 30, or 100 mg/Kg of *C. xanthocarpa* extract or 100 mg/Kg of ASA. Control animals received only vehicle. Pharmacological treatments were given orally once a day for 5 consecutive days. The last treatment was performed 3 h before the experiment. Animal body weights and general behavior were recorded at the beginning and at the end of the subacute treatment to assess the tolerability of repeated administrations.

#### 2.5.2. Bleeding

The tail transection bleeding was determined applying the method modified [20]. Briefly, tails of mice under light isofluorane anesthesia were transected at 2 mm from the tip and immersed in 1 mL of 37°C saline for 2 min. Red blood cells were lysed by adding 20 *μ*L of triton 5% and absorbance of the solution was read at 560 nm. The amount of hemorrhage was estimated by linear regression analysis of a standard curve obtained from samples of mice blood properly diluted.

#### 2.5.3. Acute Pulmonary Thromboembolism

To verify the acute pulmonary thromboembolism, a modification of method [21] was used. Pulmonary acute thromboembolism was induced in mice by rapid intravenous injection in the tail vein of a mixture of 12 mg/Kg collagen and 1 mg/Kg epinephrine in order to induce about 85% of paralysis in the control group. The loss of the righting reflex for 30 s was considered as indication of paralysis. The occurrence of paralyzed animals was recorded for 15 min after thrombotic mixture injection. Immediately after the last test, mice were euthanized by CO_2_ chamber.

#### 2.5.4. Ulcerogenic Activity

To evaluate the gastric tolerability of animals after oral administration of *C xanthocarpa *extract, they were fasted for 18 h prior to the last extract exposure (water *ad libitum*) and three hours after they were euthanized. The stomachs were opened by cutting along the greater curvature and washed with saline 4°C. Immediately after that, the development of lesions was assessed with support of a magnifying glass. The quantification of gastric mucosal lesions was scored according to their number and size in a scale from 0 up to 8 points, adapted from previous method [22], as follows: (0) without injury, (1) color modification, (2) few petechia/alterations of villous, (3) 1–3 small injuries (≤1 mm length), (4) 1–3 big injuries (≤1 mm length), (5) 1–3 big injuries (>1 mm), (6) more than three small injuries, (7) more than three big injuries, and (8) more than three deep injuries.

### 2.6. Effect on Prothrombin Time (PT) and Activated Partial Thromboplastin Time (aPTT)

For the measure of aPTT, citrate plasma (0.1 mL) was mixed with 0.1 mL of human placenta lipid extract (Pathrombin; Behringwerke), and the mixture was incubated for 2 min at 37°C. Coagulation was initiated by the addition of 0.1 mL calcium chloride (25 mM), at which point the coagulometer was started and time to clot formation was recorded [23].

For the measure of PT, citrated plasma (0.1 mL) was incubated for 1 min at 37°C, at which point 0.2 mL of human thromboplastin (Thromborel; Behringwerke) was added. The coagulometer was started, and time to clot formation was recorded [23].

### 2.7. Statistical Analysis

Results were expressed as means ± SEM, except the gastric lesion scores that were expressed as median. Values of effective concentration that induce 50% of effect (EC_50_) were reported as geometric means accompanied by their respective 95% confidence limits. *E*_max_ (maximal effect) and *I*_max_ (maximal inhibition) were calculated based on response of control groups. Data were analyzed by one-way analysis of variance (ANOVA), or *t-*test when appropriate. Post hoc tests (Student-Newman-Keuls test-SNK) were carried out when appropriate. Differences between groups were analyzed by *χ* ^2^ test for pulmonary thromboembolism. Nonparametric Kruskal-Wallis followed by Dunn's test was used to analyze gastric lesion scores. The EC_50_ values were determined by nonlinear regression analysis using a sigmoidal concentration-response equation of individual experiments using GraphPad Software 5.0 (GraphPad, USA). *P* < 0.05 was considered indicative of significant differences between groups.

## 3. Results

### 3.1. *In Vitro* Assays

**Figure 1:**
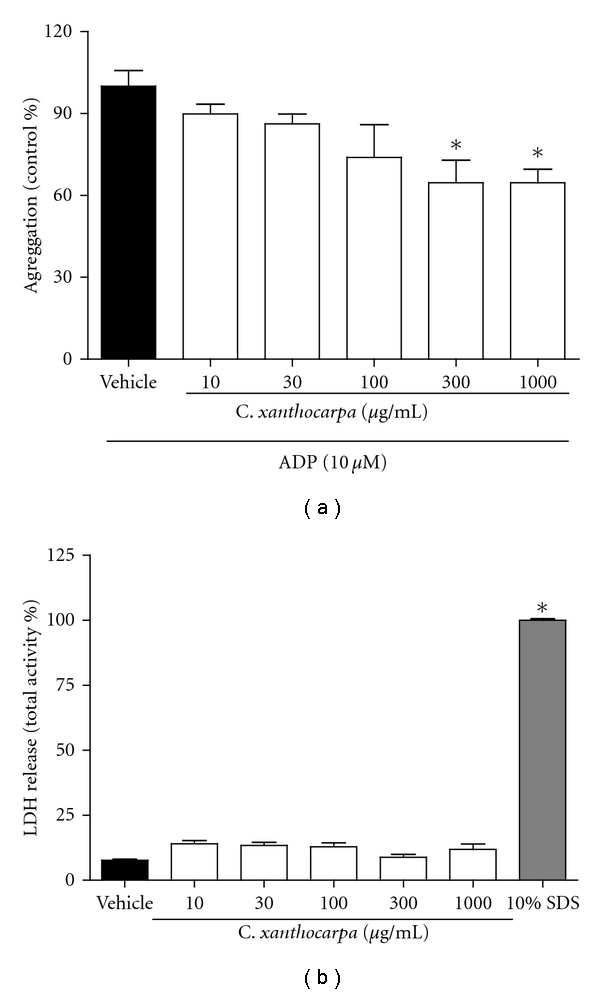
Effects of *Campomanesia xanthocarpa *extract on ADP-induced platelet aggregation (a) and on LDH release in platelets (b). Data are shown as mean ± S.E.M. (*n* = 4–6). *P < 0.05 versus vehicle control (one-way ANOVA, followed by Student Newman-Keuls' test).

We firstly evaluated the antiplatelet effects of *C. xanthocarpa *extract using freshly isolated human platelets against agonist-induced platelet aggregation. *C. xanthocarpa *extract showed a concentration-dependent inhibition of ADP-induced platelet aggregation. The *I*_max_ observed was 36 ± 5% for the concentration of 1000 *μ*g/mL and the EC_50_ value of the *C. xanthocarpa *extract was 35 (15–84) *μ*g/mL compared with the control group (Figure 1(a)).

To examine the cytotoxicity of *C. xanthocarpa *extract, we measured the LDH release from platelet for the determination of cell lysis. No concentration tested of *C. xanthocarpa *extract induced LDH release, while the positive control, SDS 10%, significantly increased the LDH release (Figure 1(b)) reflecting that *C. xanthocarpa* does not affect cell integrity. 

**Figure 2:**
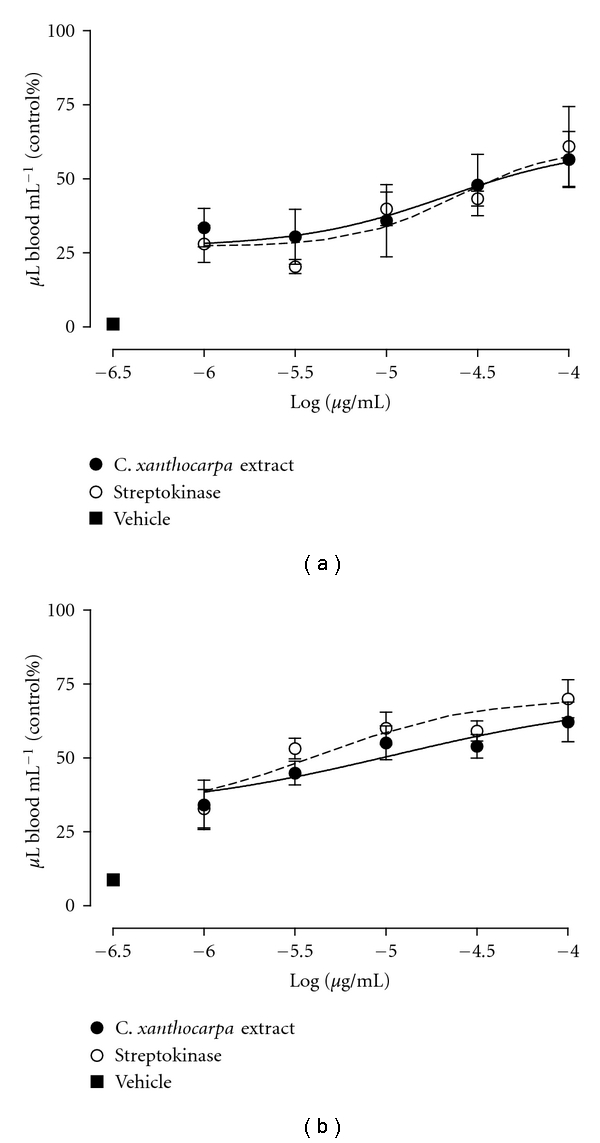
Effects of *Campomanesia xanthocarpa *extract or streptokinase on fibrinolytic activity. Concentration-response curve to mice (a) or human (b) blood. Each point represents the mean of 3 experiments performed in duplicate and the vertical lines represent SEM (nonlinear regression analysis).

In the evaluation of the fibrinolytic effect, blood clot degradation was observed in all the test tubes of *C. xanthocarpa *extract or streptokinase, with spreads of red blood cells trapped by multiple fibrins in mice and human blood. In mice blood, the EC_50_ value to *C. xanthocarpa *extract and streptokinase was 21 (5–87) *μ*g/mL and 24 (9–64) *μ*g/mL, and the *E*_max_ observed was 56 ± 9% and 60 ± 14%, respectively, for the concentration of 100 *μ*g/mL of both (Figure 2(a)). In human blood, the EC_50_ value to *C. xanthocarpa *extract and streptokinase was 11 (3–33) *μ*g/mL and 4 (1–10) *μ*g/mL, and the *E*_max_ observed was 62 ± 7% and 70 ± 6%, respectively, for the concentration of 100 *μ*g/mL of both (Figure 2(b)). 

### 3.2. *In Vivo* Assays

**Figure 3:**
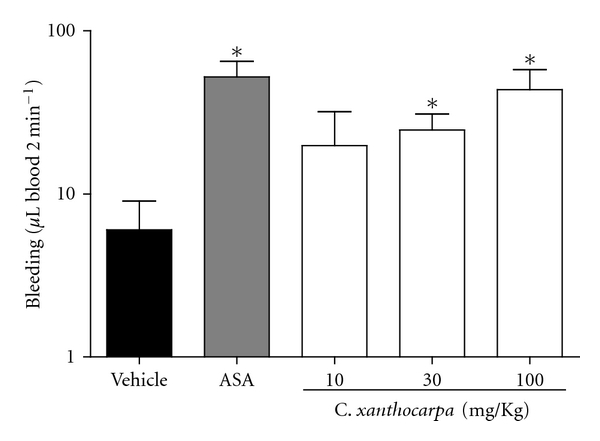
Effects of *Campomanesia xanthocarpa *extract or ASA on bleeding in mice. Data are shown as mean ± SEM (*n* = 4). *P < 0.05 versus vehicle control (one-way ANOVA, followed by Student Newman-Keuls' test).

The mean amount of blood lost from control animals 2 min after tail transaction was 5.8 ± 3.1 *μ*L. Mice treated with antithrombotic doses *C. xanthocarpa*, 30 and 100 mg/Kg/day, showed significant difference from control, losing 24.3 ± 6.6 and 43.4 ± 14.5 *μ*L of blood, respectively. ASA-treated animals lost 51.9 ± 12.9 *μ*L of blood, showing a significant difference when compared to the control group (Figure 3).

*C. xanthocarpa *had highly significant antithrombotic activity when administered at 100 mg/Kg day since it prevented paralysis induced by events in 80% compared with control. ASA displayed lower efficacy since it showed no significant difference compared to the control to reduce paralysis (Table 1). 

We also evaluated the possible ulcerogenic activity of *C. xanthocarpa* extract. None of the doses of the extract were capable of inducing ulcerogenic activity, while aspirin (100 mg/Kg/day, positive control) induced the formation of gastric lesions (the medians (25–75 percentiles)) with lesion scores of 0 (0-1); 4 (3-4); 0 (0-1), 1 (0-1), and 2 (1-2) for vehicle; aspirin: 10 mg/Kg, 30 mg/Kg, and 100 mg/Kg day^−1^ of *C. xanthocarpa* extract, respectively.

### 3.3. Effect on *Ex Vivo* aPTT and PT

As shown in Table 2, the aPTT and the PT in the vehicle group were 30.90 ± 0.25 sec and 11.33 ± 0.08 sec, respectively. In the *C. xanthocarpa*-treated groups, the aPPT increased significantly to 32.30 ± 0.91 sec and 33.13 ± 0.63 sec, at the doses of 30 and 100 mg/Kg, respectively, although remaining in normal limits when compared to the vehicle group. There were no significant increases of PT when compared to the vehicle group (Table 2).

## 4. Discussion and Conclusions

Platelets are blood cells that participate in the human primary hemostatic mechanism. Platelet-platelet interaction has the final purpose to produce a platelet thrombus that constitutes the primary hemostatic plug [24]. In addition, platelet adhesion and aggregation on blood vessel walls contribute to the occurrence of thrombosis and emboli formation, and have relation with other cardiovascular diseases [25–27]. Thus, it is important to evaluate platelet function in the thrombosis. Several studies have been carried out to develop antithrombotic agents with improved efficacy for preventing or treating arterial or venous thrombosis [28, 29]. Here, we tested the effect of *C. xanthocarpa* on platelet aggregation, as well as its antithrombotic and fibrinolytic activities that are still unknown. The results of the present study demonstrated that *C. xanthocarpa* extract has a significant protective effect in thrombosis once it is capable of inhibiting platelet aggregation without demonstrating cytotoxicity. Furthermore, *C. xanthocarpa *extract slightly prolonged aPTT, showed a fibrinolytic activity, and did not show ulcerogenic activity after oral administration.

The interactions between platelets and various adhesive proteins, such as collagen, and soluble agonists, such as ADP, provide potential targets for developing antiplatelet agents [30, 31]. The effect of *C. xanthocarpa *extract on haemostasis in *in vivo* assays was studied using a model of pulmonary thromboembolism induced by the intravenous injection of collagen plus epinephrine. This model is characterized by the massive activation of circulating platelets and the widespread formation of platelet thrombi in the microcirculation of the lungs leading to disseminated pulmonary microembolism and paralysis of the animal [32]. The oral administration of *C. xanthocarpa *extract, once a day for 5 days, markedly reduced the percentage of paralysis in a fairly dose-dependent manner. *C. xanthocarpa *extract resulted to be more effective than ASA in preventing paralysis. During thrombus formation, ADP plays a key role inducting the interaction of membrane receptor glycoprotein IIb–IIIa with fibrinogen, being the most important aggregating agent [26, 33, 34]. Thus, ADP was chosen as agonist to induce platelet aggregation in the study. Results showed that during platelet aggregation, the *C. xanthocarpa *extract significantly inhibited ADP-induced platelet aggregation. All results suggest that the antithrombotic activities of *C. xanthocarpa *extract were related at least in part to its ability to inhibit platelet aggregation. 

Blood coagulation is not only the result of a complex process initiated by the intrinsic system or the extrinsic system and/or a common pathway, but also a highly regulated process involving interactions between platelets, plasma coagulation factors, and the vessel wall [35]. Inhibitors (anticoagulants) and activators (procoagulant) of blood coagulation may affect any of the factors. The PT test and the aPTT test are used for distinguishing between the effects of test agents on the extrinsic and intrinsic pathways. In the clinical tests of blood coagulation, aPTT is used to evaluate the intrinsic clotting index. A prolonged aPTT usually represents a deficiency in factors VIII, IX, XI, XII, and V or Willebrand's factor. PT is used to evaluate the extrinsic clotting pathway. A prolonged weak PT indicates a deficiency in coagulation factors V, VII, and X [36]. The results reported here show that while the *C. xanthocarpa* did not have anticlotting effect when examined by the PT test, a slight anticoagulant effect is portrayed by the aPTT test. This indicates that the *C. xanthocarpa *might not inhibit a factor or factors in the intrinsic pathway of blood coagulation. At this stage, there is no indication of the presence of anticoagulants or procoagulants components in the *C. xanthocarpa,* since it did not affect the PT. Although *C. xanthocarpa* has shown slightly anticoagulant effect on aPTT test, this effect did not show clinical significance because the values remained in normal limits.

To further assess the possible effect of *C. xanthocarpa *on platelet activity, the bleeding in mice was measured. The evaluation of bleeding is a crude test of haemostasis, which is a useful tool to estimate how well platelets interact with blood vessel walls to form blood clots [37]. Here, we demonstrated that *C. xanthocarpa *extract treatment and ASA affect blood loss. The transactional bleeding time in mice model is sensitive not only to platelet-active compounds, but also to drugs that are capable of influencing the fibrinolytic system [38]. Moreover, *C. xanthocarpa *extract displayed favorable gastric effects and did not induce gastric lesions after oral administration up to 100 mg/Kg, demonstrating to be safer than ASA. Our results are in accordance with a previous preliminary preclinical study that showed that *C. xanthocarpa *proved to be effective in preventing gastric ulceration in rats and did not produce toxic symptoms in mice [39]. Besides, the treatment with *C. xanthocarpa* extract was well tolerated since the treated mice grew similarly to control mice (data not shown).

We also investigated the fibrinolytic activity of the *C. xanthocarpa *extract, which showed fibrinolytic activity in human and in mice compared to streptokinase. To further investigate the possible mechanism of action of *C. xanthocarpa*, streptokinase was used as standard. Probably, *C. xanthocarpa *show a same mechanism similar to the streptokinase. We suggest that the primary role of *C. xanthocarpa* is the conversion of plasminogen to plasmin. Activated plasmin enzymatically cleaves fibrin, which, with platelets and other hemostatic elements, underlies the pathological processes of the acute occlusive disorders [40]. Besides, when plasmin breaks down fibrin, a number of soluble parts are produced as the D-dimer, that is, a small protein fragment present in the blood after a blood clot is degraded by fibrinolysis [41]. Preliminary results of our group demonstrated that both *C. xanthocarpa* and streptokinase produced D-dimer during fibrinolytic activity. These optimistic results point to the value of further studies in this field. Then with the results presented in the paper we could only assume that *C. xanthocarpa* might have a similar mechanism of streptokinase, and the result suggested that the *C. xanthocarpa *extract, besides inhibiting platelet aggregation, also exerted its antithrombotic activity through fibrinolytic activity. Antithrombotic agents acting in only one pathway of thrombosis formation have limited efficacy in treating arterial thrombotic diseases [42]. Hence, the *C. xanthocarpa *extract with a combination of antiplatelet aggregation, antithrombotic, and fibrinolytic activities may be effective in preventing thrombus formation through several pathways.

Saponins are widely distributed in plants and have many biological activities, such as antiplatelet activity [14, 15]. The phytochemical components responsible for *C. xanthocarpa* antiplatelet effect are still unknown, but preliminary studies demonstrated the presence of tannins and flavonoids (quercetin, myricetin, quercitrin, and rutin) in this plant [39, 43]. Moreover, recent preliminary phytochemical analysis of *C. xanthocarpa *by our group indicated the intense presence of saponins, tannins, and terpenes and small presence of flavonoids [13]. Thus, we suggest that *C. xanthocarpa* bioactive compounds may be inhibiting platelet aggregation. 

In conclusion, the *C. xanthocarpa *extract demonstrated antiplatelet, antithrombotic, and fibrinolytic activities in mice. The antithrombotic activity of *C. xanthocarpa *extract derived probably from antiplatelet aggregation and fibrinolytic activities. The present research showed quite optimistic results pointing to the value of further studies in this field.

##  Conflicts of Interests

The authors declared that there is no conflict of interests.

## Figures and Tables

**Table 1:** Effects of *C. xanthocarpa* extract on pulmonary thrombosis in mice.

Treatment	Dose (mg/Kg)	No. of paralysed/ no. of treated mice	Protection (%)
Vehicle	—	9/10	10
*C. xanthocarpa*	100	2/10^a^	80
	30	4/9^a^	66.6
	10	7/9	33.3
Asprin	100	5/9^a^	55.5

^a^ *P* < 0.05 versus vehicle control. Data are shown as mean ± SEM (*n* = 9-10) (*χ* ^2^ test).

**Table 2:** Effect of *C. xanthocarpa* extract on *ex vivo* aPTT and PT in mice.

Treatment	Dose (mg/Kg)	aPTT (sec)	PT (sec)
Vehicle	—	30.90 ± 0.25	11.33 ± 0.08
*C. xanthocarpa*	10	30.75 ± 0.08	11.30 ± 0.10
	30	32.30 ± 0.91^a^	11.43 ± 0.13
	100	33.13 ± 0.63^a^	11.35 ± 0.08

^a^ *P* < 0.05 versus vehicle control (one-way ANOVA, followed by Student Newman-Keuls' test). Data are shown as mean ± SEM (*n* = 6).
